# Diffuse Large B‐Cell Lymphoma of the Thyroid in a Patient With Hashimoto Thyroiditis: A Diagnostic Dilemma

**DOI:** 10.1002/ccr3.71346

**Published:** 2025-10-24

**Authors:** Shravya Singh Karki, Shivam Pandey, Smriti Karki, Deepak Paudel, Chahana Pathak

**Affiliations:** ^1^ B. P. Koirala Institute of Health Sciences Dharan Nepal; ^2^ Department of Pathology B. P. Koirala Institute of Health Sciences Dharan Nepal; ^3^ Department of Otorhinolaryngology and Head & Neck Surgery B. P. Koirala Institute of Health Sciences Dharan Nepal

**Keywords:** core needle biopsy, DLBCL, FNAC, IHC, primary thyroid lymphoma

## Abstract

Primary thyroid lymphoma (PTL) is a rare malignancy, comprising 1%–5% of thyroid cancers and 2%–5% of extranodal lymphomas, with diffuse large B‐cell lymphoma (DLBCL) being the most common subtype. PTL often coexists with Hashimoto thyroiditis, leading to diagnostic delays. We report a case of a man in his early 70s who presented with a progressively enlarging, painless anterior neck mass over 2.5 years. Initial fine needle aspiration cytology (FNAC), repeated twice over 18 months, suggested lymphocytic thyroiditis. Due to persistent growth and inconclusive FNAC results, the patient underwent a right hemithyroidectomy with central neck dissection. Histopathological examination and immunohistochemistry confirmed DLBCL, activated B cell (ABC) subtype. He was started on R‐CHOP chemotherapy. FNAC, although a first‐line diagnostic tool for thyroid nodules, has a low sensitivity to PTL, especially in the context of underlying thyroiditis. Advanced diagnostic techniques such as immunohistochemistry, flow cytometry, and core needle biopsy (CNB) significantly improve accuracy but are often inaccessible in low‐resource settings. CNB offers a preserved tissue architecture and a higher diagnostic yield, reducing unnecessary surgeries. This case underscores the diagnostic limitations of FNAC in PTL and highlights the need for improved diagnostic approaches, including CNB and auxiliary tests, to ensure early and accurate diagnosis, especially in settings with limited resources.

**Summary**

- Primary thyroid lymphoma, particularly DLBCL, is often misdiagnosed by FNAC due to low sensitivity, especially with coexisting Hashimoto thyroiditis.
- Core needle biopsy, immunohistochemistry, and flow cytometry improve diagnostic precision, reducing unnecessary surgeries and enabling timely treatment.

## 1 Introduction

Primary thyroid lymphoma (PTL) is a rare malignancy, accounting for 1%–5% of all thyroid cancers and up to 2%–5% of extranodal lymphomas, with a higher prevalence in women than in men [1, 2]. It is an aggressive tumor that typically presents as a rapidly expanding neck mass with compressive symptoms [2, 3]. Almost 98% of PTLs are non‐Hodgkin B cell lymphoma, with diffuse large B‐cell lymphoma (DLBCL) being the most common subtype (60%–70%), followed by MALT lymphoma (MALTL) (10%–23%). Less common types include follicular lymphoma (~10%), small lymphocytic lymphoma (~3%), chronic lymphocytic lymphoma (~3%), and mantle cell lymphoma (~1%) [2].

Fine needle aspiration cytology (FNAC) is the first‐line diagnostic tool for thyroid nodules, often after ultrasound evaluation (USG). However, FNAC has low sensitivity for rare thyroid cancers such as PTL and anaplastic thyroid carcinoma (ATC), leading to delayed detection and unnecessary surgeries [1].

We present a case of an elderly man with a progressively enlarging neck mass initially misclassified as lymphocytic thyroiditis in FNAC. Postoperative histopathology confirmed DLBCL, underscoring the limitations of FNAC and prompting a discussion of alternative and more reliable diagnostic approaches.

## 2 Case Presentation

### 2.1 Case History and Examination

A man in his early 70s from Southeast Nepal, a nonsmoker and occasional alcohol consumer, presented to the outpatient clinic with a two‐and‐a‐half‐year history of progressive and painless anterior neck swelling. There was no history suggestive of altered thyroid hormone levels, fever, night sweats, significant weight loss, compressive symptoms, or hoarseness of voice. Additionally, there was no history of radiation exposure or a family history of cancer. His medical history included hypertension (managed with losartan 50 mg) and hypothyroidism (managed with levothyroxine 75 mcg).

On examination, a firm solitary ovoid swelling measuring ~5 × 4 cm was observed in the anterior neck, slightly to the right of the midline. It extended from the level of the cricoid cartilage above to 1 cm above the right clavicle inferiorly. The swelling moved with deglutition but did not move with tongue protrusion.

### 2.2 Differential Diagnosis, Investigations, and Treatment

**FIGURE 1:**
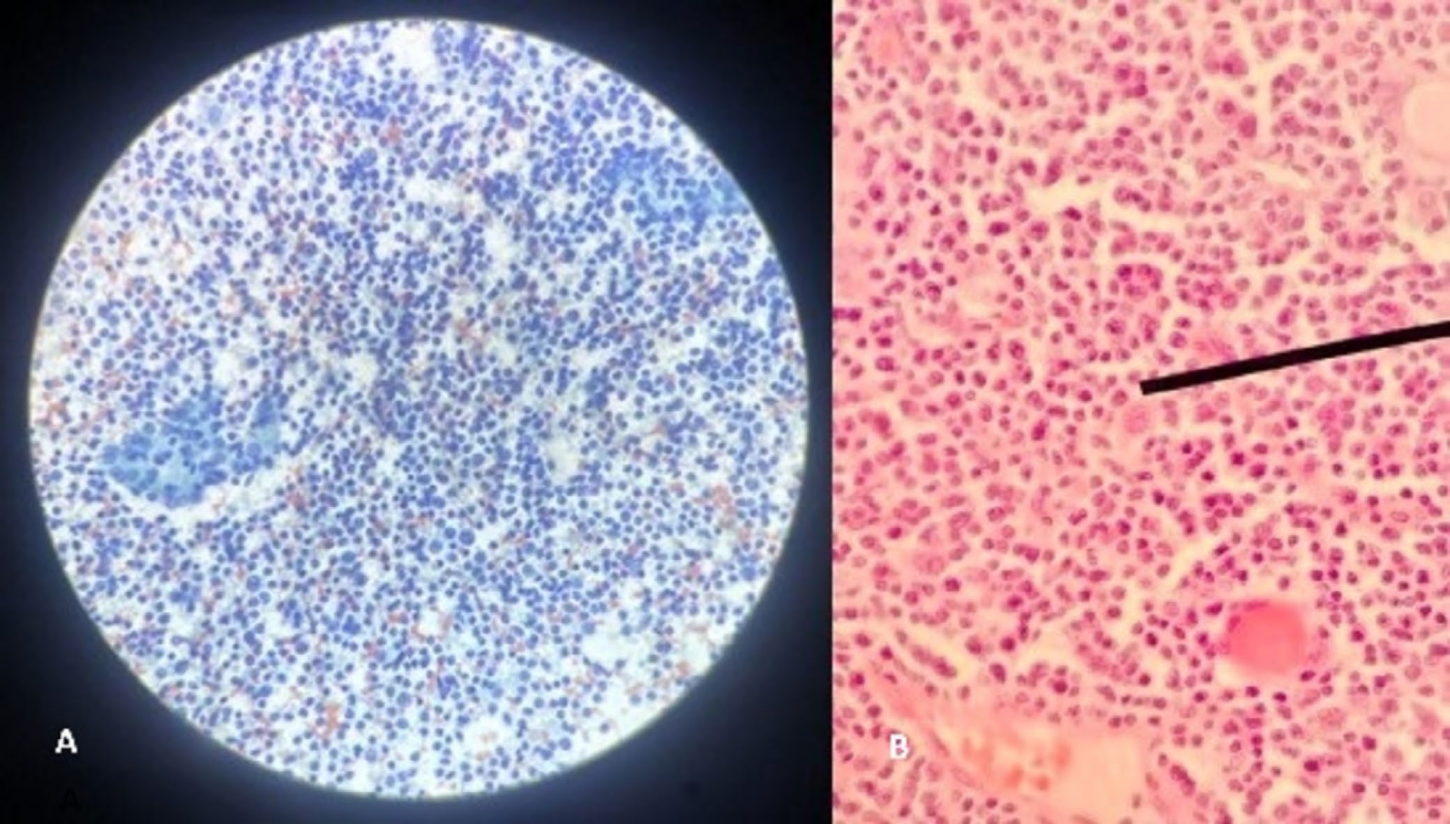
A: Diffuse proliferation of large atypical lymphoid cells (more than 2× lymphocyte size) with pleomorphic nuclei, prominent nucleoli, vesicular chromatin, and scant cytoplasm in the background of mature and transforming lymphocytes (40× Papanicolaou stain) B: Large atypical lymphoid cells with pleomorphic nuclei, prominent nucleoli, and vesicular chromatin (40×, H&E Stain).

Baseline laboratory investigations were within normal limits. Ultrasonography (USG) of the neck revealed a large, well‐defined, mixed‐echogenic mass with punctate echogenic foci in the right lobe of the thyroid. Several enlarged right cervical and prelaryngeal lymph nodes were identified, some showing loss of the fatty hilum, with the largest measuring 18.2 × 7.2 mm. Fine needle aspiration cytology (FNAC) smear showed mature and transforming lymphocytes, lympho‐histiocytes aggravating, and tangible body macrophages, and scant follicular epithelial cells against granulation tissue fragments in the background, classifying the lesion as Category II, suggestive of lymphocytic thyroiditis (Figure 1A).

Approximately 18 months earlier, the patient had similar complaints. At that time, USG revealed enlargement of both the thyroid lobes and the isthmus, with heterogeneous echotexture and increased vascularity, and the USG‐guided FNAC findings suggested lymphocytic thyroiditis. However, due to progressive enlargement and inconclusive FNAC results, the patient underwent a right hemithyroidectomy with central neck dissection.

Intraoperatively, the right thyroid lobe was markedly enlarged (11 × 7.5 cm) with solid to cystic consistency and adherence to the tracheal wall. Multiple central lymph nodes were excised, and the parathyroid glands were preserved during the procedure.

**FIGURE 2:**
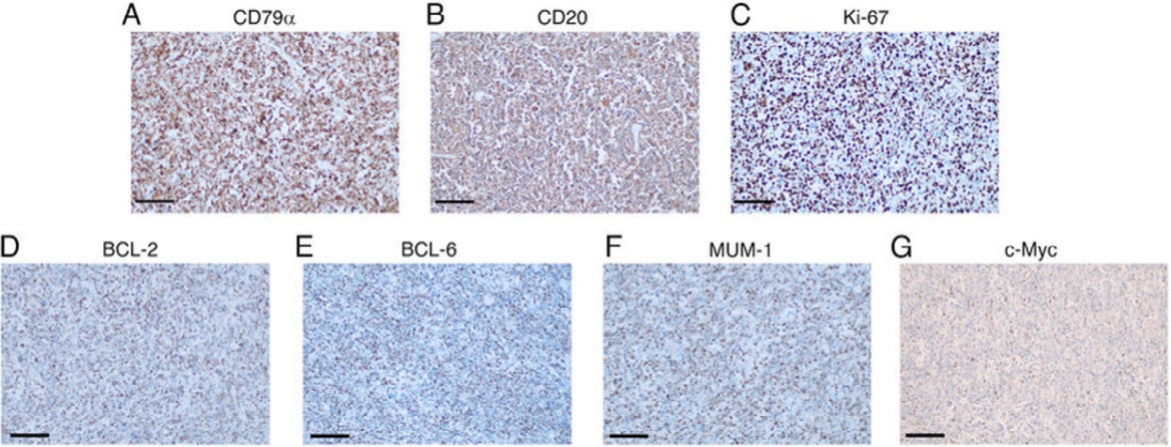
IHC staining for markers associated with DLBCL, observed under a microscope. The expression of B‐cell markers (A) CD79a and (B) CD20 is strongly positive, the expression of B‐cell lymphoma markers (C) MUM‐1, (D) BCL‐2, (E) BCL‐6, and (G) c‐Myc (≃10%) is positive, and the expression of (F) Ki‐67, a marker associated with cellular proliferation, is positive with a proliferation index of 80%. Magnification, ×200; scale bar, 60 μm. MUM‐1, mutated melanoma‐associated antigen 1. Image courtesy Jiang et al., licensed under CC BY‐NC‐ND 4.0 via https://pmc.ncbi.nlm.nih.gov/articles/PMC10722522/#abstract1.

Histopathology revealed lymphoma characteristics (Figure 1B), while IHC confirmed diffuse large B‐cell lymphoma (DLBCL), activated B‐cell (ABC) subtype. Tumor cells were positive for CD79A, BCL6, MUM1, and CD20, but negative for CD10, CYCLIN D1, EBV, CMYC, and CD30, and Ki67 proliferation index: 70% (Figure 2).

### 2.3 Outcome and Follow‐Up

Postoperative CT revealed residual recurrent lesions that surround the trachea and multiple lymph nodes in bilateral cervical, pretracheal, paratracheal, and subcarinal regions. A solid left lung nodule was also observed. The patient was diagnosed with stage IIE DLBCL (ABC subtype) and started the regimen R‐CHOP (rituximab, cyclophosphamide, doxorubicin, vincristine, prednisone). He tolerated the first cycle well and is scheduled for the second cycle in 3 weeks [4].

## 3 Discussion

FNAC is the primary invasive procedure used to assess thyroid nodules following the initial evaluation with USG. Suspicious lesions are typically subjected to FNAC, which has a sensitivity of 65%–98%, specificity of 73%–100%, false negative rates of 1%–7%, and false positive rates of 1%–11.6% [5]. However, these favorable outcomes are observed primarily in differentiated thyroid carcinomas. FNAC shows low sensitivity to detect rare tumors such as PTL [1] and ATC [6].

A systematic review by Zhang et al. reported that FNAC has a sensitivity of 48% in the diagnosis of PTL [1]. Similarly, a retrospective study by Hirokawa et al. demonstrated a specificity of 41.9%, a sensitivity of 59.4% with a positive predictive value (PPV) of 90.5% and a negative predictive value (NPV) of 94.7% in the diagnosis of PTL using FNAC [7]. Furthermore, a recent case series by Toyoshima et al. similarly demonstrated the diagnostic limitations of FNAC in primary thyroid lymphomas: in their series, FNAC yielded Bethesda VI results in all cases, yet definitive classification required a core or incisional biopsy with immunohistochemistry [8].

The low sensitivity of FNAC to the diagnosis of PTL can be partly attributed to the pathogenesis of PTL. Patients with Hashimoto thyroiditis (HT) have a 40‐ to 80‐fold increased risk of developing PTL, which typically develops 20–30 years after initial diagnosis [9]. In the background of lymphocytic thyroiditis, multiple authors have suggested that the diagnosis of MALTL is more difficult due to the presence of a large number of heterogeneous cells. On the contrary, the diagnosis of DLBCL is easier due to the presence of larger, monotonous lymphocytes.

In this case, although follicular epithelial cells were present, fine‐needle aspiration cytology (FNAC) could not reliably exclude lymphoma. This underscores the importance of correlation with clinical and imaging findings and, when needed, proceeding with core needle biopsy or histologic confirmation.

In recent decades, the sensitivity of FNAC in the detection of primary thyroid lymphomas (PTLs) has improved, largely due to the integrated use of additional techniques such as immunohistochemistry (IHC) and flow cytometry (FC). Mitsuyoshi et al. reported that FC using CD45 and side scatter‐based gating, together with G‐banding chromosomal analysis and immunoglobulin heavy chain (JH) DNA rearrangement analysis, achieved a specificity of 88.4% and a sensitivity of 75.0% in the diagnosis of PTL [7].

In IHC, PTLs typically express the leukocyte common antigen (LCA/CD45), CD79a, MS4A1 (CD20), and λ light chains, while lacking cytokeratin expression. Furthermore, DLBCL can be distinguished from MALTL, the second most common differential for thyroid lymphomas, by its higher Ki‐67 proliferation index [10]. However, FNAC samples are often deemed insufficient for these ancillary tests.

Furthermore, IHC can help determine the cell of origin, classifying DLBCL as: [10]

- Germinal Center B cell (GCB) (CD10 + or BCL6+ and IRF4 / MUM1−)
- Nongerminal center B cell/activated B cell (ABC) subtype (IRF4/MUM1+)

In our case, the IHC profile was consistent with diffuse large B‐cell lymphoma (DLBCL), the activated B‐cell (ABC) subtype, which is associated with a poorer prognosis.

Thus, USG‐guided core needle biopsy (CNB) has emerged as a superior technique for diagnosing PTL. Unlike FNAC, which disrupts tissue architecture, core biopsy preserves the structural integrity of the sample, allowing for more accurate histopathological differentiation. Additionally, higher tissue yield facilitates better IHC, FC, and genetic analysis, enhancing diagnostic precision [3, 9]. A meta‐analysis by Vander Poorten et al. found that CNB has a higher diagnostic value, with a sensitivity and PPV of 94.3% and 100% for PTL, and 80.1% and 100% for ATC. Furthermore, CNB reduces the need for diagnostic surgery to 6.2% for PTL and 17.6% for ATC, compared to 34% and 37.9%, respectively, when relying on FNAC [3, 11].

Chronic autoimmune thyroiditis, especially HT, is the primary risk factor for primary thyroid lymphomas (PTLs); hence, biochemical tests offer limited diagnostic value. Typical findings include hypothyroid profiles (elevated TSH, low T3/T4) and raised anti‐Tg or anti‐TPO levels in Hashimoto thyroiditis [12]. However, anti‐Tg is a nonspecific marker for malignancy in thyroid nodules [13].

In resource‐limited settings like ours, surgical excision biopsy remains the preferred diagnostic method despite its association with increased surgical complications and its lack of role in the treatment of DLBCL.

The modified Ann Arbor classification for primary thyroid lymphoma (PTL) categorizes the disease into four stages. Stage IE indicates confined localized disease in the thyroid gland without lymph node involvement. Stage IIE involves the thyroid and regional lymph nodes (e.g., cervical or mediastinal) on the same side of the diaphragm, similar to this case. Stage IIIE represents advanced disease with involvement of the thyroid, lymph nodes, and organs on both sides of the diaphragm (e.g., abdominal nodes or spleen). Stage IV denotes disseminated lymphoma with widespread involvement of multiple extranodal sites (e.g., bone marrow, liver, lungs). The designation “E” highlights the thyroid as an additional nodal site, and staging is typically determined by imaging, biopsy, and sometimes bone marrow evaluation [2].

Due to the rarity of the disease, no consensus has been obtained on the management of the disease. Thyroid DLBCL, an aggressive PTL, is typically managed with a combination of chemotherapy and radiation therapy, offering superior results compared to single‐modal treatments. The standard chemotherapy regimen is R‐CHOP, often followed by involved field radiotherapy (IFRT) covering the thyroid and regional neck lymph nodes, or extended field radiotherapy (EFRT) that includes the upper mediastinal nodes. Unlike stage IE MALT lymphoma, where single‐modal treatments such as surgical resection or radiation therapy may suffice, DLBCL and higher‐stage lymphomas require combined modality treatment for optimal outcomes. In advanced cases with tracheal invasion, a multidisciplinary approach incorporating R‐CHOP and internal tracheal stenting is recommended [14].

The role of surgery in managing PTL is debated. A 1992 Mayo Clinic study found that aggressive debulking surgery did not provide a benefit over open biopsy to diagnose advanced PTL when complete resection was not feasible, with open biopsy followed by chemotherapy showing a slightly higher response rate (88% vs. 85% for debulking with adjuvant chemotherapy) [15]. Minimally invasive diagnostics, such as CNB and FC, have further reduced the need for surgical biopsies in the management of PTL. When PTL is diagnosed after thyroidectomy—often due to preoperative misdiagnosis as benign or neoplastic—adjuvant chemoradiation is typically needed to prevent recurrence [14]. Aggressive thyroid surgery has risks, including damage to recurrent laryngeal nerves, trachea, esophagus, major vessels, and parathyroid glands, making it less viable. However, Alfonso et al. and Fatima et al. suggest that surgery may relieve compressive symptoms in advanced thyroid DLBCL [16, 17].

In conclusion, this case highlights the diagnostic challenge of primary thyroid lymphoma (PTL) in the context of Hashimoto's thyroiditis, especially when fine‐needle aspiration cytology (FNAC) yields equivocal or borderline findings. Although cytology remains a valuable tool, its limitations in detecting lymphoma—due to sampling error, poor image quality, or low cellularity—must be acknowledged. The addition of ancillary techniques (such as immunohistochemistry, cell block analysis, and flow cytometry) and consideration of core needle biopsy (CNB) should be part of the diagnostic pathway in suspicious or rapidly enlarging thyroid masses.

Clinically, recognizing the possibility of PTL early is critical, as prompt histological subclassification informs the most effective therapeutic strategy, which may involve combinations of surgery, chemotherapy, and radiotherapy. This case also underscores the importance of making accurate diagnostic decisions in settings with limited resources, where advanced ancillary techniques may not be readily available. Future work should aim to standardize image acquisition and reporting (including cytologic image quality), assess the role of antibody serology where feasible, and explore decision algorithms to guide when CNB is warranted over or in addition to FNAC.

## Author Contributions

**Shravya Singh Karki:** conceptualization, formal analysis, supervision, validation, writing – original draft. **Shivam Pandey:** conceptualization, formal analysis, writing – original draft. **Deepak Paudel:** resources, supervision, writing – review and editing. **Smriti Karki:** investigation, resources, supervision, writing – review and editing. **Chahana Pathak:** writing – review and editing.

## Ethics Statement

The patient and the patient party were explained the use of their clinical scenario and images in a depersonalized form for report writing and publication purposes, both verbally and followed by obtaining written consent for the same. Clearance from the Institutional Review Committee (IRC) approval is not required to publish a case report at our institute.

## Consent

Written consent obtained from the patient covered consent for publication.

## Conflicts of Interest

The authors declare no conflicts of interest.

## Data Availability

Data sharing not applicable to this article as no datasets were generated or analysed during the current study.
